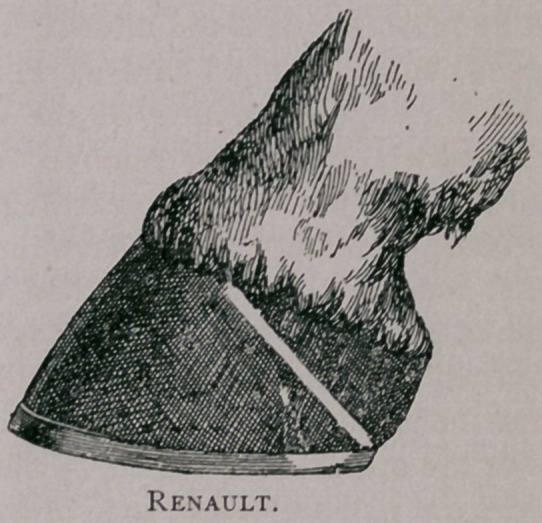# Quittor or Quitter

**Published:** 1892-02

**Authors:** Charles Williams


### QUITTOR OR QUITTER (*Webster*).

By Charles Williams, V. M. D.

Synonyms.—*Fesselgeschwür*, German ; *Giarda*, Italian ; *Gialarrs.* Spanish ; *Javart*, French.

The etymology of the word is entirely unknown, it being a term by which old veterinary writers have described various affections of the lower extremities of the horse, donkey, mule, and even bovines. There is a mortification and softening of the structures, and an elimination of the dead parts by slough and suppuration. In several old works these sloughs are called Quittors (javars), till it has gradually settled onto the disease itself. Vatel strenuously opposed the name, but, notwithstanding that, it is the term generally applied amongst hippopathologists. Girard divided them into four classes or divisions : (1st) simple or cutaneous, which is only the furuncle which occurs in the true derm structure nearest the coronary band. (2d) The tendinous, which closely resembles the felon in man where the subcutaneous connective tissue, as well the tendon itself, becomes a victim to the inflammatory process and sloughs away. (3d) The subhorny or quittor of the coronary band, this is a furuncle of the cutidura of the coronary band, and the slough involves the superior portion of the laminated tissue. (4) cartilaginous, or the caries of the lateral fibro-cartilaginous prolongations of the os pedis, which old writers confounded with that of the coronary band; true, it may be a complication of the former, or the two may exist at the same time.

Taking them up in the order mentioned, the first and simplest, the *cutaneous quittor*, is simply a furuncle of the coronary region of the foot, on account of the thickness and elasticity of the derm in this region, resulting in strangulation of the inflamed tissues beneath by the very painful compression. The hind feet are more subject to it than the fore, and it is more frequent at the heels in the flexion of the fetlock, but it may occur on the sides and front of the coronet, in which case it is much more painful. It is also seen in cattle ; but in these it is usually associated with the tendinous variety, and then the bones and articulations are affected thus producing a true felon as found in man.

Symptoms.—Cutaneous Quittor is first characterized by a tense but painful swelling of the coronary region. The color, of course, is not changed, except in unpigmented hides, in which case it is red or of a livid hue. The disease is accompanied with a diffuse œdema extending to the fetlock or even to the hock or knee. The lameness is extreme, the animal scarcely resting on the diseased member, and in some cases even producing constitutional results, as fever, loss of appetite, etc., etc. After the tumor reaches a certain size it softens at the summit, the base remaining hard, and it finally ulcerates and discharges a small quantity of bloody pus, followed by the appearance of a slough. An abscess is now formed in the tumor. The slough forms a kind of core, which remains attached by its base for some time, and when at last loosened and coming away, leaves a round, deep cavity, extending through the tumor from top to bottom. As soon as this takes place the lameness generally subsides, and if there are no complications the wound readily heals.

Complications.—Cutaneous quittor is often complicated by tendinous, if the process of destruction extends to the underlying structures. This complication is especially common in cattle, and the disease is generally more severe in them, and gives rise to more swelling and greater pain than in the horse. The course is usually short, lasting from one to two weeks unless complicated.

Etiology.—The causes of cutaneous quittor are bruises, but punctured wounds are frequent causes. But there may be no apparent cause. Mud, manure, and filth, impregnated with urine, in which animals may have to stand and be exposed in cold weather, are causes, hence the disease is more prevalent in winter than in summer. The mud of cities seems to be more irritating as it often contains salt, lime, and other mineral and chemical substances, which are discharged into the gutters from manufacturing establishments, dye houses, etc., etc. Calcareous soils are said to produce it, and last, but not least, clipping and the parasites of the Symbiotic mange (Chiroptis-Spathiferus), which only attacks the legs of horses. Coarse lymphatic animals with hairy legs are predisposed, and then when these animals have their legs clipped in the barbarous and senseless manner now so common (for it is a fact that a skin protected with long hair is more delicate than one covered by a short coat), hence, when deprived of its natural covering, together with the irritation of the stubby ends of the shorn hairs, it becomes irritated, cracked, and inflamed, and finally develops the disease above named.

Treatment.—The treatment consists in lancing the parts quickly, or even resorting to deep scarifications, to allow the escape of the pent-up serum, and thus prevent sloughs by the death of large quantities of tissue by strangulation. The suppurative process may be hastened by a blister or poultice or both, when required, and after healthy granulations are set up it may be treated as a simple wound.

*Tendinous Quittor*, Syn. Hornwurme (*Germ.*) Zundel considers it an analogue of felon, but this is obviously a mistake, as a true felon starts in the deep structures, in the bone itself, even under the periosteum. Tendinous quittor is often a complication of the cutaneous form, it attacks the tendons, and especially the flexors. and often the ligaments, and even the bones themselves may subsequently become affected producing periostitis, and even synovitis and arthritis.

The symptoms are vague at first. There is excessive lameness, even though there is no visible change in the leg itself, the animal suffers intensely ; but the symptoms are not sufficiently marked or definite to be able to locate it definitely and positively.

The foot is indicated as being affected by the animal lifting it quickly and high, and placing it carefully in walking, or carrying it altogether. In two (2) to five (5) days, a tumor hot and painful appears at the coronet above the heel, even more painful than in the cutaneous form. The limb may swell to the knee or hock, and the animal show all the symptoms of severe fibrile disturbance ; appetite lost, rapid breathing, mucous membranes injected, thirsty, and often occupying the recumbent position, except when it is located in the hind limb. The process of suppuration is not as well localized, and the diagnosis of the one or more abscesses is always difficult, especially when situated in the very deep tissues. After opening we do not get a simple wound as in the cutaneous, but a persistent fistula extending deep down to a necrosed portion of tendon. Sometimes, almost at the start, we find numerous small papules in the fold of the fetlock, which end in as many deep fistulæ which discharge a thick fœtid humor, which is often mixed with blood. This disease in cattle is usually more painful even than in the horse, and always accompanied by extensive swelling even to the carpus or hock. The compulsory resting of the limb and consequent overtaxing of the other may produce complications of laminitis or breaking down. The diagnosis is at first obscure, till symptoms are all well marked. The prognosis is grave on account of the deep lesions being often complicated with the articular structures, thus causing deformity or loss of function, or both. The cause of this trouble originates in about the same manner as the preceding: blows, treads, bruises, punctures, etc., etc., irritating muds, complications of cutaneous, and Fischer says it may complicate distemper. The treatment consists of evacuating the pus if formed, and if not, deep scarifications to allow the escape of the pent-up serum and blood which often engorges the inflamed parts. The diseased parts should be removed as much as possible and the parts gotten healthy, and then dressed antiseptically and as a simple wound.

*Cartilaginous Quittor.*—" Fibro Chondritis of the third phalanx (*Vatel*) Peculiar to solipeds on account of their only having lateral cartilages.

These fibro-cartilages are two pieces, one on either side of the heel, which, with the plantar cushion, complete the base of the heels.

They are parallelogram in shape, the external face is convex, the internal is concave, they extend back of the navicular bone, and are pierced by numerous foraminæ for the passage of blood-vessels, nerves, etc. They are separated from the skin by connective tissue and a rich vascular network. The internal network is hollowed by vascular grooves and covers the articular borsa forward. They are attached before and below to the retrossal and basilar processes, and anteriorly to the anterior lateral ligament by a fibrous band, and closely to the ant[illegible]rior lateral ligament. In structure they are not pure cartilage, but are largely mixed with fibrous tissue. They are more developed in the forefeet than in the hind, and the internal is often higher than the external.

Cartilaginous Quittor is a serious disease characterized by partial caries of one of these fibro-cartilages. It is a partial gangrene, whose character is to spread slowly. This disease is usually seen in heavy horses on account of their peculiar work, causing bruises, blows, treads, in fact any wound that complicates or exposes these lateral cartilages. Work in stone quarries, lumber yards, ship-yards, wharves, etc., etc., predispose to the trouble The disease is more common in cities than in the country. Flat feet with low heels are most often affected. The disease may complicate suppurating corns or street nails, and interfering is a very fruitful cause.

The symptoms vary with the nature and situation of the disease. Those in the posterior zone are less sensitive than in the anterior. The advance of the disease and depth of the fistula, together with disposition of the animal, determine the amount of lameness. Well-bred animals are always more sensitive to pain than the cold bred carters. There is always more or less tumefaction, which is often indurated, with one or more fistulous tracks entering its substance.

Zundel divides the symptoms into the Acute and Chronic. In the acute form we have all the symptoms of active inflammation. There is always much fever in the part with more or less œdema, and with intense pain of a lancinating character. The skin may or may not be compromised at the beginning. The intense pain is due to the sensitiveness of the parts because of the close proximity to the terminal filaments of the sensory nerves, and is much increased by the pressure of the œdema under the unyeilding skin and horn. These symptoms continue and even increase till suppuration is established and the pus has found an exit, when it may be temporarily relieved, only to be repeated with the formation of new abscesses as the disease advances.

In the chronic form the symptoms are less marked. There may be an indolent ulcer on the coronet with slight œdema, and much less pain in the preceding, except when of long standing and the disease has invaded the deep tissues. After a quittor is well established there will be found a large ovid lump on the coronet, generally on the inside, with an ugly, livid, granulating wound. There is usually much discharge for the size of the wound, which is usually fœtid and of an oily consistency, yellowish-white in color, and on examination and manipulation the granulations bleed freely, and on exploration there will be found one or more fistulous tracks penetrating to variable depths, which often communicate, or they may be entirely distinct. The disease progresses steadily forward and never backward, and in long standing cases that have started posteriorly, a series of cicatrices, with the active fistula in front, may be seen. This is explained by the cartilage being diseased in regular course, and as it sloughs away the pus is discharged, but as the other tissues are more vascular and able to withstand the action of the pus, the fistula closes, leaving the pus to accumulate and burst out at another place more directly over the seat of the disease. The constant irritation of the coronary band by the action of the pus and the fever of the parts, causes the hoof to grow rough, undulated and thickened.

On examination the cartilage always manifests lesions proportionate to the duration of the disease. The cartilage presents a darkened line, and at the point of caries a greenish hue surrounded with a dull, dark, reddish zone of necrosis. The fistula is only the vacuum, so to speak, or the vacated part once occupied by cartilage; it is lined by a sort of pyogenic membrane caused by the action of the pus on the adjacent cells.

Renault, and after him, Lafosse, mention a certain change taking place in the cartilage, probably caused by the acid pent-up secretions, similar to the action of acid on bone whereby they are softened and thus approach the embryonal state. The cartilage undergoing this change becomes yellowish in color, and yields easily under pressure of the finger or allows a probe to enter it readily. The above writers say the proper way to diagnose this change is to either thin the horn or simply elevate the coronary band when the cartilage can be examined. Lafosse says, in this stage it is not necessary to remove the whole cartilage, but simply to excise the necrosed portion, or to stimulate the sloughing process by the so-called caustic treatment, hence the success of the empyrics that use this treatment.

When one considers the situation and nature of the disease it hardly seems possible that spontaneous recovery should ever take place, but, wonderful to relate, it does, but in rare instances, for ordinarily the disease progresses, slowly but surely, destroying the cartilage, and finally ends up by opening the articulation and even extending till the ligaments and tendon of the anterior extensor are involved, and even the bones may become necrosed and septic infection occur in long standing cases.

The diagnosis is made on the foregoing symptoms, the œdema of the coronet with fistulous tracks, discharging large quantities of fœtid yellowish pus, often mixed with blood.

The prognosis is always grave, not merely from the danger of loss by death of the animal, but from the expense and loss of time necessary to accomplish a cure. And, if as in old cases, we may get the disease so far advanced as to be beyond our power to check its progress till large structural changes have taken place. When there are already such complications as open joint, abscess in joint, or other important structures are involved in the diseased process, it will be advisable to destroy the animal at once, unless it be a very valuable brood animal.

When the disease is acute, simply thinning the horn over the region of the enlargement and soaking, poulticing and allowing the escape of the pus through a dependent opening, together with a blister over the whole, may be all that is necessary. But if the incrosis is well established, we will have to have recourse to more heroic measures, several of which are in vogue; such as the actual and potential cautery and the removal of the cartilage. In the treatment by the actual cautery, the parts are simply laid open and the diseased parts cauterized, which is only admissible when the disease is situated posteriorly ; but it is often a source of aggravation, and only augments rather than cures the trouble. In using the potential cautery, Solleysel principally recommended Hy. cl., mixed with aloes. This was also mentioned by Girard, Bernard and others, among them many English veterinarians, who used the drugs in the solid form, and in this way did not get the rapid results to be obtained by the use of liquid caustics, and they often destroyed the surrounding healthy tissues or irritated them to such extent as to increase the trouble as much as by the actual cautery before mentioned. Zundel prefers liquid caustics, as he says they modify the process of decomposition, dry up the suppurative process, and stimulate the healthy tissues without injuring them. This treatment is credited to Mariage in 1847, and he used Valette's solution, others have used 10 per cent. solution, of bichloride of mercury, nirtate of silver, per chloride of iron, chloride of copper and zinc, etc. It is difficult, according to Zundel, to decide which are preferable, and he says we all can confirm his judgment, "*none* are infallible," and it depends more upon the constancy in treatment than upon the drugs employed. To obtain a cure by the caustic treatment, it is necessary that the application be made every day and several times a day with a syringe, and the fistulas (it is insisted by all the advocates) should be enlarged so that there may be free drainage for the escape of all liquids. Some even make dependent openings through the thinned walls ; some place wooden plugs in the fistulas to distend their walls ; others plegits of oakum wet with Vallette's solution ; while others still have recourse to setons. During this treatment it is urged the animals should *not* be worked, and if the caustic should irritate greatly, a poultice should be applied, or the foot soaked, and a carefully fitted shoe, to protect the quarter without injuring it, will be of advantage. When the treatment is successful, the discharge changes to a more laudable nature, and is much increased, the granulations take on a better color, and finally the discharge decreases, and at last the injections are made with difficulty and the wound closes. But the danger of this mode of treatment is, of injuring some of the deeper structures, especially of opening the joint, for while we are treating the disease from without, we are totally ignorant of what is taking place within, for the wound may even appear to do well and close, only to break again further forward, and every time nearer the joint, and other structures that we so much fear to injure. We then have the same process to renew again, with the increased risk of permanently injuring the animal. Thus we should weigh well our chances of success by waiting longer, or have recourse at once to the *third method* of treatment, viz. :

Removal of the cartilage.—This operation was first recommended, it is said, by Lafosse, Sr., 1754, and was often performed by his son. Zundel has rightly said it is one or the most valuable results of the application of anatomical knowledge to the practice of veterinary surgery. Bourgelat, Girard, Hurtel Darbroval and many others have also performed it and written of it. Zundel says (*to my surprise*) that it has little favor in England, Germany and even in France, and is only resorted to after the caustic treatment has been tried and failed. It is an operation of great delicacy, and accompanied with great risk on account of the close proximity to the articulation between the second and third phalanx, and it requires at once a thorongh anatomical knowledge, together with a careful, steady and experimental hand, to insure satisfactory results. We must have a care not to injure the coronary band or the podophylous tissue, both of which are essential to the reorganization and growth of healthy horn. The lateral ligament of the joint must also be avoided, and last, and above all, the synovial membrane before mentioned, which lies directly beneath the cartilage, and in certain rare instances has been known to be imbedded into the internal face of the cartilage; this probably takes place in old and hard-worked animals with distended bursæ, thus causing resumption of the cartilage by continued pressure. The cartilage may be removed entirely or in part. In the latter only a portion of the fibro cartilage is remmoved. Vatel, Sanstus, Renault, Bell and Lafosse have reported many cases of recovery by this mode, and I have also had success in a single case. Zundel thinks that it will not be successful unless accompanied by as favorable circumstances as would lead to recovery from the caustic treatment, and he thinks, and I quite agree with him, that when once the operation is begun, to thoroughly complete it rather than incur the risk and annoyance of having to perform a second operation. The operation of removal of the whole cartilage includes two principal steps : First, the removal of the horn of wall and sole, corresponding to the diseased part and as far forward as the end or attachment of the cartilage anteriorly ; and second, the removal of the cartilage. The opinions of surgeons vary as to the amount of horn to remove. But it is generally agreed that the superior border shall extend from the anterior extremity of the cartilage backward ; that is, the two posterior thirds of space from toe to heel, or one-third of the circumference of the coronary band. Opinion varies as to the lower border. Lafosse, Sr., drew the line downward, parallel with the fibres of the horn, and thus made the

La Fosse, Sr.

La Fosse, Jr.

lower border longer than the upper, and so compromised the plantar surface too much and made much more work for the operator (a very important matter).

Lafosse, Jr., went to the other extreme by not extending his to the sole, and removed only a cresent-shaped zone below the operation. Renault crossed the fibres and brings the line down to

Rey.

Renault.

the sole one-half the distance of the upper border. While Rey prefers to run the line almost perpendicular to the ground surface. I believe, most operators agree that Lafosse, Sr., cuts too much, and his son too little, while Renault probably is the best of all, because, while he removes enough to allow of a successful operation, it does not remove so much of the sole as to compromise the bearing of the foot, and in any other way to weaken the foot unnecessarily, and thus prolong the illness of the animal. The foot should be thoroughly soaked or poulticed for at least twenty-four hours before the operation to insure its softening, for diseased feet, one knows from experience, become dry and hard from the accompanying fever. The hair on the coronet should be thoroughly cleansed and clipped short. The sole and bars corresponding to the part, as well as the wall, should be thinned to the thickness of writing paper before casting, and thus shorten the time required to keep the animal confined in the recumbent position. The animal should then have administered something to blunt the sensibility to pain. I prefer chloral hydrate to all others, in from one to two ounces in a draught. The horse should then be immediately cast on a good, soft bed, and the limb put in position for the operation, the details of which it is unnecessary to review.

The advisability of giving an anæsthetic is twofold : First, from a humanitarian standpoint; and second, it expedites the operation by quieting the animal, and also decreases, in my opinion, the risk of a broken back or other injuries consequent to the violent exertions of an unanesthetised animal. The operation commenced, we thin the coronary region carefully and evenly, taking care not to injure the underlying living tissues, but it must be so thin as to offer as little resistance as possible to the knife while cutting through and lifting the coronary band, which is the second step ; this is done by means of a sharp sage-knife, and must be done with great care and dispatch, so as not to allow any mishap, caused by the struggles of the animal, to injure the coronary band, for that once injured, a diseased and faulty growth of horn is inevitable.

Zundel, instead of this method of thinning the wall, recommends the barbarous method of cutting through the wall around the edge of the parts corresponding to the operation, and then prying off and stripping it from its podophyllous structure; our objections to this are obvious : first, it is so cruel; and secondly, there is a large raw surface uncovered, together with the exposed ends of innumerable torn nerves to increase the pain and complicate the healing process. Before lifting the coronary band, a twitch made out of a broad, strong piece of burlap should be placed round the pastion and twisted tight with an inserted horseshoe, to prevent the excessive hemorrhage which follows opening up the parts. In making the incision, in order to turn back the coronary band, I have adopted a method belonging to a friend, Dr. C. H. Magill, of this city, namely, of inserting the knife nearly one-quarter of an inch below the darkened line of the coronary band, and thus leaving more vital substance with the band, and thus preventing the tendency to slough from want of nourishment. The knife inserted, care must be taken to keep the edge turned slightly downward so as to cut clean against the cartilage, and thus leave the loose connective tissue together with the nerves and vessels intact, and attached to the skin to support its life and hasten the ultimate recovery of the wound. We insert the knife at the anterior extremity of the cartilage with the cutting edge backward; it is then carried backward to the posterior border of the cartilage and down into the cleft of the heel ; then with the back taken as an axis, the edge is rotated downward, forward, and upward, till the whole posterior portion or wing is excised or removed, the remaining portion is then removed piecemeal, to avoid the underlying structures. A precaution that is enjoined at this point is to strongly extend the foot while working over the bursa so that it may be drawn tense and thus avoid injury. It is better to leave a thin flake of cartilage over the bursa, than in our anxiety to get every piece, to open the capsule. When all is removed carefully a "quittor shoe" (a shoe with one-quarter widened till it covers the whole diseased quarter of the diseased foot) is applied, always leaving one nail out at the toe opposite the diseased quarter so that a large nail may be driven in it to form a hook to hold a portion of the bandages. Then the process of dressing is to begin by first cleansing the part thoroughly from blood and foreign matter, and when thoroughly dry I am very particular to cover every portion with Tr. Benzoin Comp., and then dust liberally with a powder composed of Iodoform 1, Tannic Acid 3 parts. The oakum should be thoroughly picked and free from all knots and foreign bodies, and the whole cavity then filled with the loose picked flakes till the coronary band is held in about the natural position, not too full, and yet full enough to bear moderate pressure. The oakum is now applied in large elongated pads, thicker in the middle and tapering to the edges, so as to make a dressing about the shape of half a lemon. The bandages should be made of some strong material, such as unbleached muslin, or ticking torn in strips 1½ inches wide by 5 yards long. The muslin is good enough till the animal begins work, and is much cheaper, thus avoiding the temptation to use a bandage a second time.

The application is made first at the inferior portion and tied in the opposite heel, and with an assistant to hold the free end, the rolled portion is then passed round from before to behind and at the top of the dressing and with the third turn passing over the convexity. Then it should begin again at the bottom and progress regularly upward, lapping over the preceding one about one-half, thus having no free border looking upward to be trodden downward ; the last turn should always finish above the convexity ; the free ends of the bandage should now be drawn under the heel bandage in opposite directions and tied, the dressing fastened and the tourniquet removed ; the animal may be allowed to rise, and if the operation is carefully done and the dressing properly adjusted, you will often see him stand on the sore foot immediately.

The dressing should be left about three to four days without removal, when it will be found necessary to remove it and replace with fresh materials and antiseptics, after which it will be necessary to dress it only when the dressings become very foul, or the patient becomes restive and uncomfortable.

If everything progresses favorably and without complication the animal should be sufficiently recovered to begin moderate work in from four to six weeks.

The complications that may occur are : 1, opening the joint ; 2, necrosis of anterior lateral ligament ; 3, caries of os. pedis, 4, alteration coronary band, etc. The joint may be opened during operation or may slough through afterward, in either case it is not so severe as might be supposed, provided their no complication of of suppuration going on within. A paste of bichloride of mercury with starch or flour, or a touch of silver nitrate, with firm pressure, is all that is usually required to complete the cure.

I and my friend, Dr. Magill, operated on a mule when suppurative arthritis had already set in, and while removing a portion of cartilage posterior, but *not over* the bursa, the membrane ruptured spontaneously, and a large gush of purulent synovia was the result. We, of course, told the owner the consequent prognosis and the case was abandoned by him, but we kept the animal to note the result, which was perfect recovery from the operation ; the mule has a low ring bone and occluded joint, but is a very useful animal on soft ground. In the other complications the diseased portions should be removed and carefully dressed with antiseptics or resolvents, and they should be of little consequence.

## Figures and Tables

**Figure f1:**
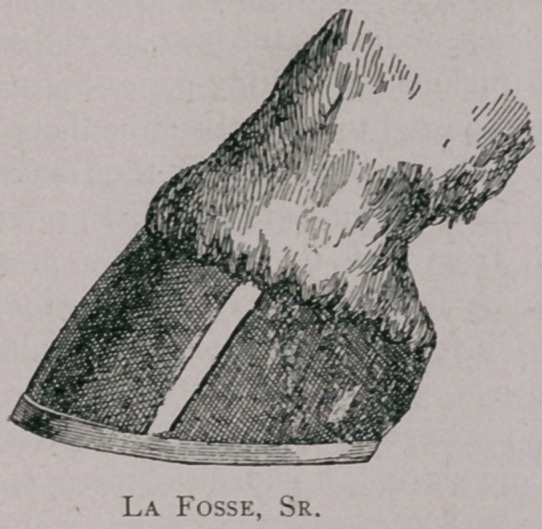


**Figure f2:**
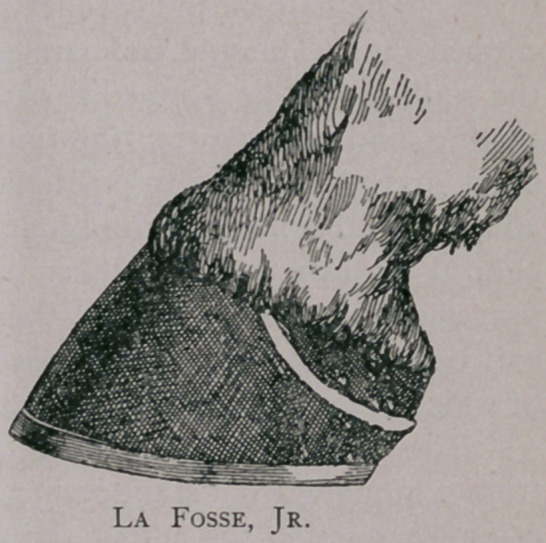


**Figure f3:**
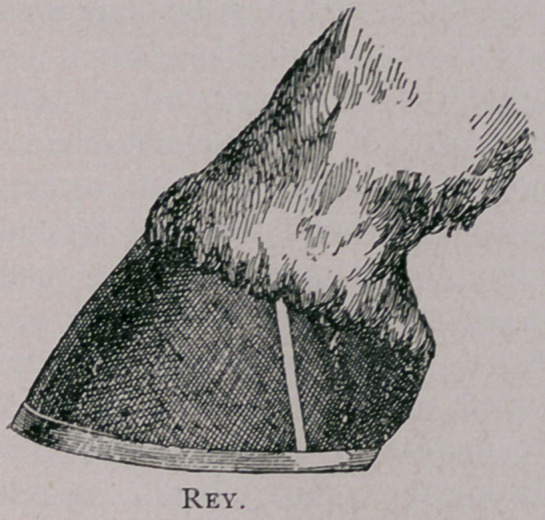


**Figure f4:**